# Hypertension‐related morbidity after delivery discharge: A comparison of nifedipine versus labetalol

**DOI:** 10.1002/pmf2.70357

**Published:** 2026-07-02

**Authors:** Sonya Fabricant, Matthew Cervantes, Glenda Marshall, Natalie Bello, Emily Seet, Sarah Kilpatrick, Mariam Naqvi

**Affiliations:** ^1^ Department of Obstetrics & Gynecology Cedars‐Sinai Health Sciences University Los Angeles California USA; ^2^ Department of Cardiology Cedars‐Sinai Health Sciences University Los Angeles California USA

**Keywords:** hypertension, maternal morbidity, postpartum, preeclampsia

## Abstract

**Objective:**

To evaluate rates of hypertension‐related morbidity after delivery discharge among individuals on nifedipine versus labetalol monotherapy.

**Methods:**

This was a retrospective cohort study of individuals who delivered at a level IV obstetric hospital from January 1, 2021 to January 20, 2024, had a hypertensive disorder of pregnancy, and were discharged on nifedipine or labetalol monotherapy. The primary outcome was hypertension‐related post‐discharge morbidity, defined as any of the following within 30 days of delivery discharge: severe hypertension, de novo preeclampsia lab abnormalities, or new‐onset clinical morbidity (hemolysis, elevated liver enzymes and low platelets [HELLP] syndrome, eclampsia, pulmonary edema/acute heart failure, cerebrovascular disorders, myocardial infarction/cardiac arrest, liver hematoma, or acute renal failure). Secondary outcomes included hypertension‐related hospital return and hospital return length of stay. Data were abstracted from the medical record. Logistic regression and bivariate analysis were used to compare groups. Multivariable logistic regression was used to adjust for baseline characteristics and readmission risk factors from the literature.

**Results:**

Of 360 patients meeting inclusion criteria, 67.2% were discharged on nifedipine and 32.8% were discharged on labetalol. Nifedipine recipients had lower body mass index and were less likely to have chronic hypertension compared to labetalol recipients. Post‐discharge morbidity occurred less frequently among individuals discharged on nifedipine compared to labetalol (1.7% vs. 10.2%; adjusted odds ratio [aOR], 0.1; 95% confidence interval [CI], 0.05–0.5). Individuals on nifedipine were less likely to return to the hospital in the 30 days following discharge (4.6% vs. 12.7%; aOR, 0.3; 95% CI, 0.1–0.7) and less likely to have a hospital return lasting ≥24 h (1.7% vs. 6.8%; OR, 0.2; 95% CI, 0.07–0.8).

**Conclusion:**

Among individuals on antihypertensive monotherapy at delivery discharge, nifedipine was associated with significantly reduced odds of post‐discharge morbidity compared to labetalol.

## 1 INTRODUCTION

Hypertensive disorders of pregnancy (HDPs), including gestational hypertension, preeclampsia, eclampsia, and chronic hypertension, complicate approximately 7%–10% of pregnancies and remain a leading cause of adverse maternal outcomes in the postpartum period [1, 2, 3]. Blood pressure rises postpartum, peaking between 3 and 7 days after delivery, a period when many patients have already been discharged from the hospital [4, 5]. Optimizing hypertension management during this critical window is essential to reducing adverse maternal health outcomes [6].

Nifedipine and labetalol, both first‐line antihypertensive agents in pregnancy, are two of the most commonly prescribed medications to control blood pressure in the antepartum and postpartum periods [7, 8]. Prior studies comparing the two medications concluded that nifedipine was associated with lower risk of postpartum hypertension‐related readmission when compared to labetalol [9, 10, 11, 12, 13]. Less is known, however, regarding how discharge antihypertensive medication affects other adverse maternal outcomes [10, 12, 13]. Improved understanding of the impact of antihypertensive type on postpartum outcomes beyond readmission can help guide clinicians in optimizing care for their patients [14].

We therefore sought to compare rates of hypertension‐related clinical morbidity after delivery discharge between individuals on nifedipine versus labetalol monotherapy. We hypothesized that exposure to nifedipine, compared to labetalol, would be associated with a reduction in post‐discharge hypertension‐related morbidity (severe hypertension, preeclampsia‐related lab abnormalities, and/or clinical morbidity). This hypothesis was based on the diuretic and natriuretic effects of nifedipine and its potential to mitigate the fluid shifts seen in the postpartum period [15, 16]. Secondarily, we examined the association between discharge antihypertensive type and risk of postpartum hypertension‐related hospital return.

## 2 MATERIALS AND METHODS

**FIGURE 1:**
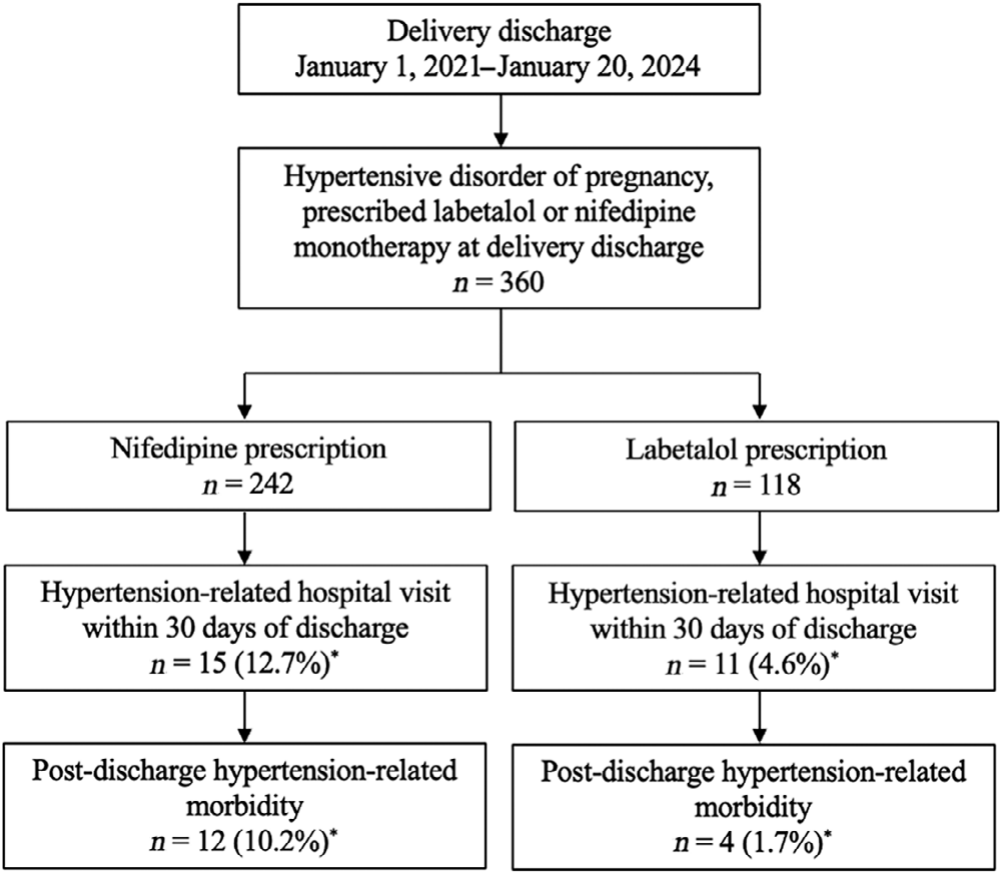
Flow diagram of study population. *Includes any of the following: severe hypertension (systolic blood pressure ≥160 mmHg or diastolic blood pressure ≥110 mmHg obtained in the hospital setting), de novo preeclampsia lab abnormalities (aspartate aminotransferase [AST] ≥ 68 U/L, alanine aminotransferase [ALT] ≥ 110 U/L, serum creatinine > 1.1 mg/dL, or platelet count <100,000 cells/µL), or new‐onset clinical morbidity (hemolysis, elevated liver enzymes and low platelets [HELLP] syndrome, eclampsia, pulmonary edema/acute heart failure, stroke/cerebrovascular disorders, liver capsule hematoma or rupture, or acute renal failure).

This was a retrospective cohort study of all deliveries at a level IV obstetric hospital over a 3‐year period (January 1, 2021 to–January 20, 2024) (Figure 1). Individuals were included in the study if they (1) had a diagnosis of HDP during delivery admission and (2) were prescribed either labetalol or extended‐release nifedipine monotherapy at delivery discharge. Individuals on more than one antihypertensive at discharge were excluded. Those meeting eligibility criteria were identified by Cedar‐Sinai Medical Center's Enterprise Information Service's (EIS) Honest Enterprise Research Brokers (HERB).

Our primary outcome was post‐discharge hypertension‐related morbidity, defined as any of the following in the 30 days after delivery discharge: severe hypertension (any systolic blood pressure ≥160 mmHg or diastolic blood pressure ≥110 mmHg obtained in the hospital setting), de novo preeclampsia lab abnormalities (aspartate aminotransferase [AST] ≥ 68 U/L, alanine aminotransferase [ALT] ≥ 110 U/L, serum creatinine >1.1 mg/dL, or platelet count <100,000 cells/µL), or new‐onset clinical morbidity (hemolysis, elevated liver enzymes and low platelets [HELLP] syndrome, eclampsia, pulmonary edema/acute heart failure, stroke/cerebrovascular disorders, liver capsule hematoma or rupture, or acute renal failure). To meet criteria for the primary outcome, the individual must not have had the outcome present during their delivery admission (with the exception of severe hypertension, as this was typically the indication for initiation of antihypertensive therapy), and the outcome had to be attributable to an HDP based on medical record review. For example, an individual with HDP and a serum creatinine >1.1 mg/dL during their delivery admission who was subsequently re‐admitted with an elevated serum creatinine would not meet criteria for the primary outcome.

Secondary outcomes included any hypertension‐related hospital return within 30 days of delivery discharge (including emergency department visits, obstetric (OB) triage visits, and hospital readmissions) and the hypertension‐related hospital return length of stay. A hypertension‐related hospital return included any hospital visit for elevated blood pressure, preeclampsia‐related symptoms, or preeclampsia lab abnormalities. As our institution lacked a standardized protocol for postpartum home blood pressure monitoring during the study period, post‐discharge outcomes were limited to those identified at the time of hospital return.

The postpartum blood pressure treatment threshold at our institution was 140/90 mmHg. Antihypertensive therapy was initiated with one of the following agents and starting doses: extended‐release nifedipine 30 mg once daily, labetalol 200 mg twice daily, or enalapril 2.5 mg twice daily. Agent selection and subsequent titration to achieve target blood pressure were left to the discretion of the obstetric attending physician. Similarly, decisions regarding discharge antihypertensive regimen, outpatient blood pressure monitoring, and post‐discharge management were made at the discretion of the treating clinician.

Baseline and obstetric characteristics, vital signs, laboratory values, and readmission status were extracted from the medical record by HERB. Additional variables including HDP diagnosis at delivery discharge, medical comorbidities, antihypertensive medication type and dose at delivery discharge, and details regarding readmission outcomes were obtained by manual chart review and abstraction. Medical record review was performed by two trained clinicians. Outcomes were classified as hypertension‐related based on study staff review of chart documentation by the treating clinician at the time of the clinical event.

Baseline clinical and obstetric characteristics were compared between the nifedipine and labetalol groups using Fisher's exact test, independent sample *t*‐test, or Mann–Whitney U test where appropriate. For categorical outcome variables, odds ratios (OR) with 95% confidence intervals (CIs) and *p* values from logistic regression are reported; for those outcomes with zero events, *p* values from Fisher's exact test are reported. Multivariable logistic regression was used to adjust for maternal age ≥40 years, delivery body mass index (BMI) ≥35 kg/m^2^, and insurance type. These confounders were chosen based on their documented association with hypertension‐related readmission in the existing literature [17, 18].

A sensitivity analysis was performed in which severe hypertension was excluded from the composite morbidity outcome to determine whether observed associations were robust when restricting the definition to laboratory or clinical morbidity events. Additionally, as baseline chronic hypertension may impact both the type of antihypertensive prescribed and the risk for adverse peripartum outcomes among individuals with HDP, a subgroup analysis excluding individuals with chronic hypertension was performed for the primary outcome [19, 20].

Statistical analysis was performed using StataNow/SE 18.5 (StataCorp LLC). A *p* value <0.05 was considered statistically significant. This study was approved by the Institutional Review Board at Cedars‐Sinai Medical Center. Informed consent was waived due to the retrospective nature of this study.

## 3 RESULTS

During the study period (January 1, 2021 to January 20, 2024), 360 individuals were discharged on nifedipine or labetalol monotherapy and had a diagnosis of an HDP on chart review. Of these, 67.2% were discharged on nifedipine monotherapy while 32.8% were discharged on labetalol monotherapy. Clinical characteristics by discharge antihypertensive type are presented in Table 1. Those on labetalol had a higher median BMI and were more likely to have baseline chronic hypertension (with or without superimposed preeclampsia). HDP subtype otherwise did not differ significantly between groups. Individuals on nifedipine had higher maximum systolic and diastolic blood pressures from delivery to discharge but had a lower maximum systolic blood pressure in the 24 h prior to discharge (Table 1). A similar proportion of patients in both groups were discharged on the lowest dose of their given antihypertensive agent (labetalol 200 mg twice daily: 65.3% vs. nifedipine 30 mg daily: 59.5%, *p *= 0.3). There were no patients in either the nifedipine or the labetalol group who were prescribed furosemide at delivery discharge. Remaining clinical characteristics from delivery admission were similar between groups.

**TABLE 1:** Baseline characteristics among individuals on nifedipine versus labetalol monotherapy at delivery discharge.

Variable	Labetalol (*n* = 118)	Nifedipine (*n* = 242)	*p*
Age (years)	36 [33–40]	35 [33–40]	0.9
Public insurance	19 (16.1)	28 (11.6)	0.2
Race			
Asian (non‐Hispanic)	22 (18.8)	38 (15.8)	0.8
Black (non‐Hispanic)	18 (15.4)	37 (15.4)	
Hispanic	26 (22.2)	46 (19.1)	
White (non‐Hispanic)	43 (36.8)	101 (41.9)	
None of the above or unknown	8 (6.8)	19 (7.9)	
BMI at delivery (kg/m^2^)	32.7 [29.3–36.8]	30.1 [27.1–34.8]	<0.001
Tobacco use	1 (0.9)	4 (1.7)	1.0
Pregestational diabetes	7 (5.9)	10 (4.1)	0.4
Gestational diabetes	15 (12.7)	29 (12.0)	0.9
Pre‐existing renal disease	2 (1.7)	1 (0.4)	0.3
Chronic hypertension	43 (36.4)	36 (14.9)	<0.001
Multifetal gestation	6 (5.1)	19 (7.9)	0.4
In vitro fertilization	15 (12.7)	36 (14.9)	0.6
Antenatal aspirin prescription	56 (47.5)	96 (39.3)	0.1
Gestational age at delivery (weeks)	37.8 [35.7–39]	37.4 [35.9–39]	0.97
Nulliparity	61 (51.7)	147 (60.7)	0.1
Cesarean delivery	72 (61.0)	139 (57.4)	0.6
Hypertensive disorder diagnosis at delivery discharge			
Chronic hypertension alone	12 (10.2)	9 (3.7)	0.06
Gestational hypertension	11 (9.3)	14 (5.8)	
Preeclampsia without severe features	5 (4.2)	8 (3.3)	
Preeclampsia with severe features	88 (74.6)	203 (83.9)	
HELLP syndrome	1 (0.9)	7 (2.9)	
Eclampsia	1 (0.9)	1 (0.4)	
Maximum SBP, delivery to discharge (mmHg)	160 [153–170]	166 [158–177]	<0.001
Maximum DBP, delivery to discharge (mmHg)	94 [89–101]	97 [93–104]	0.005
Maximum SBP within 24h of discharge (mmHg)	149 [142–155]	144.5 [138–153]	0.01
Maximum DBP within 24h of discharge (mmHg)	89 [83–92]	88 [84–92]	0.9
Severe hypertension in the 24 h prior to discharge^a^	16 (13.6)	23 (9.5)	0.3
Postpartum hospital length of stay (days)	3.8 [3–5.1]	4.1 [3.1–5.1]	0.7
Prescribed furosemide at discharge	0	0	–
Prescribed lowest dose of given antihypertensive at discharge^b^	77 (65.3)	144 (59.5)	0.3

*Note*: Data presented as *n* (%) or median [IQR].

Abbreviations: BMI, body mass index; DBP, diastolic blood pressure; HELLP, hemolysis, elevated liver enzymes and low platelets; SBP, systolic blood pressure.

^a^ Blood pressure ≥ 160/110 mmHg.

^b^ Labetalol ≤200 mg twice daily, Nifedipine ≤30 mg/day.

Hypertension‐related morbidity after delivery discharge was less common among those discharged on nifedipine, occurring in 1.7% of the nifedipine group compared with 10.2% of the labetalol group (OR, 0.1; 95% CI, 0.05–0.5; Table 2). Considering the individual components of the primary outcome, severe hypertension occurred significantly less frequently in the nifedipine group compared to the labetalol group (1.7% vs. 9.3%; OR, 0.2; 95% CI, 0.05–0.5). In the labetalol group, there were two cases of de novo preeclampsia lab abnormalities and two cases of pulmonary edema/acute heart failure. No additional new‐onset clinical morbidities were identified in either group. In a sensitivity analysis removing severe hypertension from the composite primary outcome, nifedipine monotherapy remained significantly associated with lower post‐discharge morbidity compared with labetalol (0% vs. 3.4%, *p *= 0.01) (Table 4).

**TABLE 2:** Hypertension‐related morbidity after delivery discharge among individuals on nifedipine versus labetalol monotherapy.

Outcome	Labetalol (*n* = 118)	Nifedipine (*n* = 242)	Odds ratio (95% CI) or *p* value as applicable
Primary outcome: composite post‐discharge morbidity^a^	12 (10.2)	4 (1.7)	0.1 (0.05–0.5)
Severe hypertension^b^	11 (9.3)	4 (1.7)	0.2 (0.05–0.5)
Preeclampsia lab abnormalities^c^, ^d^	2 (1.7)	0	0.1
Clinical morbidity^d^	2 (1.7)	0	0.1
HELLP syndrome	0	0	–
Eclampsia	0	0	–
Pulmonary edema/acute heart failure	2 (1.7)	0	0.1
Stroke/cerebrovascular disorder	0	0	–
Myocardial infarction/cardiac arrest	0	0	–
Liver capsule hematoma/rupture	0	0	–
Acute renal failure	0	0	–
Secondary outcomes			
Hypertension‐related hospital return	15 (12.7)	11 (4.6)	0.3 (0.1–0.7)
Hospital return, not readmitted	5 (4.2)	6 (2.5)	0.6 (0.2–1.9)
Hospital readmission	10 (8.5)	5 (2.1)	0.2 (0.08–0.7)
Delivery to readmission (days)	8.6 [5.7–11.3]	5.9 [3.9–8.2]	0.4
Hospital return length of stay (days)	1.1 [0.2–2.1]	0.2 [0.1–1.4]	0.2
Hospital return length of stay ≥ 24h	8 (6.8)	4 (1.7)	0.2 (0.07–0.8)
Maximum SBP during readmission (mmHg)	172 [147–189]	154 [144–175]	0.2
Maximum DBP during readmission (mmHg)	95 [83–99]	88 [86–95]	0.2

*Note*: Data presented as *n* (%) or median [IQR]. *p* values for outcomes with calculable odds ratios are derived from the Wald test from logistic regression. *p* values for outcomes with zero events in one group are derived from Fisher's exact test.

Abbreviations: CI, confidence interval; DBP, diastolic blood pressure; HELLP, hemolysis, elevated liver enzymes and low platelets; SBP, systolic blood pressure.

^a^ Includes any of the listed outcomes within 30 days of delivery discharge.

^b^ Any blood pressure ≥160/110 mmHg during hospital return.

^c^ Maximum AST ≥ 68 U/L, maximum ALT ≥ 110 U/L, maximum serum creatinine >1.1 mg/dL, minimum platelet count <100,000.

^d^ New‐onset during post‐discharge hospital return.

Multivariable logistic regression for the primary outcome was performed, adjusting for established risk factors for postpartum readmission among individuals with HDP (age ≥ 40 years, BMI ≥ 35 kg/m^2^, and insurance type) [17, 18, 21, 22]. Nifedipine remained significantly associated with a reduced odds of post‐discharge hypertension‐related morbidity (adjusted odds ratio [aOR], 0.1; 95% CI, 0.05–0.5; Table 3).

**TABLE 3:** Logistic regression model for maternal outcomes after delivery discharge among individuals on nifedipine versus labetalol monotherapy.

Variable	Adjusted odds ratio^a^ (95% CI)	*p*
Morbidity following delivery discharge^b^, ^c^	0.1 (0.05–0.5)	0.001
Hypertension‐related hospital return^b^	0.3 (0.1–0.7)	0.005

Abbreviation: CI, confidence interval.

^a^ Controlled for age ≥40 years, delivery body mass index ≥35 kg/m^2^ and public insurance.

^b^ Within 30 days of delivery discharge.

^c^ Any of the following: severe hypertension (any systolic blood pressure ≥160 mmHg or diastolic blood pressure ≥110 mmHg obtained in the hospital setting), de novo preeclampsia lab abnormalities (AST ≥ 68 U/L, ALT ≥ 110 U/L, serum creatinine >1.1 mg/dL, or platelet count <100,000 cells/µL), or new‐onset clinical morbidity (hemolysis, elevated liver enzymes and low platelets [HELLP] syndrome, eclampsia, pulmonary edema/acute heart failure, stroke/cerebrovascular disorders, liver capsule hematoma or rupture, or acute renal failure).

Individuals discharged on nifedipine compared to those on labetalol were less likely to return to the hospital for a hypertension‐related indication within 30 days of delivery discharge (4.6% vs. 12.7%; OR, 0.3; 95% CI, 0.1–0.7; Table 2). Similarly, those on nifedipine were less likely to be readmitted for a hypertension‐related indication (2.1% vs. 8.5%; OR, 0.2; 95% CI, 0.08–0.7), and less likely to have a hospital return length of stay of 24 h or longer compared to those on labetalol (1.7% vs. 6.8%; OR, 0.2; 95% CI, 0.07–0.8). Rates of hospital return without readmission were not significantly different (2.5% vs. 4.2%; OR, 0.6; 95% CI, 0.2–1.9). After adjusting for confounders, nifedipine prescription at discharge remained associated with significantly lower odds of 30‐day post‐discharge hospital return compared to those on labetalol (aOR, 0.3; 95% CI, 0.1–0.7; Table 3).

In the planned subgroup analysis excluding individuals with baseline chronic hypertension (*n* = 281), hypertension‐related morbidity was lower with nifedipine compared with labetalol (1.5% vs. 10.7%; OR, 0.1; 95% CI, 0.04–0.5) (Table 4). Hypertension‐related hospital return also remained lower with nifedipine (3.4% vs. 14.7%; OR, 0.2; 95% CI, 0.08–0.6).

**TABLE 4:** Sensitivity and subgroup analyses for primary and secondary outcomes.

Outcome	Labetalol	Nifedipine	Odds ratio (95% CI) or *p* value as applicable
Sensitivity analysis: Excluding patients meeting criteria for primary outcome solely by severe hypertension on hospital return (*n* = 360)			
Hypertension‐related morbidity	4/118 (3.4)	0/242 (0)	0.01
Subgroup analysis: Excluding patients with chronic hypertension (*n* = 281)			
Hypertension‐related morbidity	8/75 (10.7)	3/206 (1.5)	0.1 (0.03–0.5)
Hypertension‐related hospital return	11/75 (14.7)	7/206 (3.4)	0.2 (0.08–0.6)

*Note*: Data presented as *n* (%). *p* values for outcomes with calculable odds ratios are derived from the Wald test from logistic regression. *p* values for outcomes with zero events in one group are derived from Fisher's exact test.

Abbreviation: CI, confidence interval.

## 4 DISCUSSION

Nifedipine monotherapy at delivery discharge, compared with labetalol monotherapy, was associated with significantly lower rates of hypertension‐related morbidity after delivery discharge. This finding included reductions in severe hypertension, new‐onset preeclampsia lab abnormalities, and new‐onset clinical morbidity, and remained significant when excluding cases of severe hypertension alone.

We also found reduced rates of hypertension‐related hospital return with nifedipine compared to labetalol, consistent with prior research demonstrating that nifedipine use is associated with significantly lower odds of postpartum readmission [10, 11, 12, 13]. This evidence is supported by a recent randomized controlled trial in which 323 patients were randomized to nifedipine 30 mg twice daily or labetalol 200 mg three times daily [12]. In the intention‐to‐treat analysis, nifedipine was associated with reduced odds of hospital readmission (1.2% vs. 8.1%; aOR, 0.1; 95% CI, 0.02–0.56) [12]. Notably, prior studies have focused exclusively on postpartum readmission [10, 11, 12, 13]. Our study extends this literature by demonstrating that nifedipine use is also associated with lower rates of adverse postpartum clinical morbidity, providing additional insight into differences in maternal outcomes between the therapies.

Our lower rate of adverse events with nifedipine may be explained by its diuretic and natriuretic effects. Normal postpartum physiology involves mobilization of fluid from the extravascular to intravascular compartment, contributing to the typical rise of blood pressure in the postpartum period [7]. This intravascular volume shift can precipitate worsening hypertension, endothelial dysfunction, and their sequelae [1]. Nifedipine, a calcium channel blocker, increases renal blood flow and promotes both diuresis and natriuresis through its effects on the proximal convoluted tubules, thereby blunting the expected postpartum rise in blood pressure [15, 16].

It has also been proposed that the once‐daily dosing option of nifedipine may improve medication adherence over labetalol, which requires more frequent (twice or three times daily) dosing [11]. However, one randomized controlled trial comparing once versus twice daily dosing of nifedipine found no difference in need for antihypertensive dose increase in the postpartum period [23]. The remaining research investigating the relationship between once and twice daily dosing of nifedipine with postpartum outcomes remains limited, and it is unclear if dosing frequency alone can account for the observed differences in postpartum outcomes.

Nifedipine (a calcium channel blocker) and labetalol (an alpha and beta blocker) are both considered first‐line agents in the treatment of pregnancy‐related and postpartum hypertension [5]. Both are compatible with lactation and have unique but comparable side effect profiles [8]. Among nonpregnant adults, calcium channel blockers are among the first‐line agents for management of chronic hypertension; beta‐blockers are not recommended as a first‐line agent but can be considered as an alternative [24]. Our findings contribute to the growing body of evidence suggesting that nifedipine may be the preferred medication over labetalol in the postpartum population with regard to maternal health outcomes and hospital resource utilization [9].

This study has several limitations. Its retrospective design raises the possibility of selection bias, reflected in baseline differences in BMI and rates of chronic hypertension between cohorts. Medication adherence could not be assessed from discharge prescriptions alone, and patient factors influencing antihypertensive selection, such as side effects necessitating an agent change, were not captured. Similarly, we cannot exclude the possibility that providers with different practice patterns may also preferentially select different antihypertensive agents for their patients, independently influencing clinical outcomes. Although our findings persisted after adjustment for baseline characteristics and in analyses excluding individuals with chronic hypertension, residual confounding cannot be excluded. Future studies with larger cohorts should consider propensity score matching to more rigorously account for residual confounding. Additionally, postpartum events occurring outside our hospital system may not have been identified given our reliance on a single institution's clinical documentation. Finally, we acknowledge that severe hypertension may not represent significant maternal morbidity in all cases. Our sensitivity analysis excluding severe hypertension from the primary outcome identified a statistically significant association between nifedipine prescription and reduced maternal morbidity; however, the small number of events in the labetalol group limits the statistical stability of this finding. Our small sample size similarly limited our ability to evaluate extremely uncommon outcomes such as cerebrovascular disorders. Our results should thus be interpreted with caution, and validation in larger prospective studies is needed.

Our study also has several notable strengths. We used a detailed medical record review rather than relying on administrative coding to ascertain medical comorbidities, delivery admission HDP diagnosis, and postpartum clinical outcomes. This approach avoids the limitations of administrative datasets, which have variable sensitivity and specificity for HDP diagnosis and have a poor positive predictive value for maternal morbidity in general [25, 26]. Our use of patient‐level data allowed us to accurately estimate rates of adverse clinical outcomes and to more precisely control for baseline characteristics. In addition, few retrospective studies have evaluated antihypertensive dosing between treatment groups. We found that similar proportions of patients were discharged on the lowest dose of their respective medication, suggesting comparable complexity in achieving hypertension control between groups. Finally, by limiting outcomes to hypertension‐related events, we were able to more precisely estimate the relationship between antihypertensive type and both primary and secondary outcomes.

## 5 CONCLUSION

Among individuals on antihypertensive monotherapy at delivery discharge, nifedipine was associated with substantially lower odds of clinical morbidity after delivery discharge compared to labetalol. These findings suggest that nifedipine may offer clinical advantages over labetalol in postpartum patients by reducing postpartum maternal morbidity. Further prospective studies are needed to confirm these findings.

## AUTHOR CONTRIBUTIONS

**Sonya Fabricant**: Conceptualization; investigation; writing—original draft; methodology; writing—review and editing; formal analysis; project administration; data curation; supervision; software. **Matthew Cervantes**: Writing—review and editing; investigation. **Glenda Marshall**: Investigation; writing—review and editing. **Natalie Bello**: Conceptualization; investigation; writing—review and editing; resources. **Emily Seet**: Conceptualization; investigation; writing—review and editing; resources. **Sarah Kilpatrick**: Conceptualization; investigation; writing—review and editing; supervision; resources. **Mariam Naqvi**: Conceptualization; investigation; writing—review and editing; supervision; resources; software; validation.

## CONFLICT OF INTEREST STATEMENT

The authors declare no conflicts of interest.

## FUNDING INFORMATION

The authors received no specific funding for this work.

## ETHICS STATEMENT

This study was approved by the Institutional Review Board at Cedars‐Sinai Medical Center (IRB # 00003594).

## Data Availability

The data that support the findings of this study are available from the corresponding author upon reasonable request.
